# Innovative Pharmacological/Therapeutic Approaches against Hepatic Ischemia/Reperfusion Injury

**DOI:** 10.1155/2015/918583

**Published:** 2015-12-21

**Authors:** Mariapia Vairetti, Hartmut Jaeschke, Diethard Monbaliu, Jae-Sung Kim, Andrea Ferrigno

**Affiliations:** ^1^Department of Internal Medicine and Therapeutics, Pharmacology and Toxicology Unit, Via Ferrata 9A, 27100 Pavia, Italy; ^2^Department of Pharmacology, Toxicology & Therapeutics, University of Kansas Medical Center, 3901 Rainbow Boulevard, MS 1018, Kansas City, KS 66160, USA; ^3^Department of Abdominal Transplant Surgery and Transplant Coordination, University Hospitals Leuven and Department of Microbiology and Immunology, University of Leuven, 3000 Leuven, Belgium; ^4^Department of Surgery, Pharmacology and Therapeutics and Institute on Aging, University of Florida, 1600 SW Archer Road, Gainesville, FL 32610, USA

Prolonged oxygen deprivation corresponding to the ischemic period is observed during liver resection, transplantation, and trauma and subsequent oxygen restoration always leads to reperfusion injury. In particular, the ischemic period represents an inevitable event during conventional organ preservation before transplantation and the magnitude and severity of reperfusion injury depend on the time of liver preservation. Faced with the increasing shortage of donor organs for transplantation, there is renewed interest in marginal livers, such as those obtained from donation after circulatory death or fatty livers, which are particularly susceptible to ischemia/reperfusion (I/R) injury.

The decreased tolerance towards I/R observed in fatty livers has not been fully elucidated yet, and underlying mechanisms appear different in lean and steatotic livers. The paper of A. Matsuda et al. proposed the use of activated protein C (APC), an anticoagulant, found to induce an initial attenuation of tissue damage by inhibiting inflammatory cell infiltration and sinusoidal endothelial injury in normal liver. Of interest, in steatotic mice livers, APC did not affect initial liver damage but attenuated late damage, suggesting the existence of an additional pathway to the anti-inflammatory cytoprotective effect of APC which may occur via activation of adenosine monophosphate-activated protein kinase (AMPK) phosphorylation.

A further attempt to reduce the fatty liver susceptibility was reported by E. Pantazi et al. that used a peroxisome proliferator-activated receptor *α* (PPAR*α*) agonist WY-14643. PPAR*α* is a nuclear receptor highly expressed in liver and it functions as a lipid sensor. Treatment with WY-14643 reduced liver injury in fatty rat livers, enhanced the deacetylase enzyme sirtuin 1 (SIRT1), recently found to be a target for preventing I/R, and prevented endoplasmic reticulum stress.

M. Bejaoui et al. investigated the use of polyethylene glycols (PEGs) that, besides their usefulness as oncotic agents in preservation solution, have been shown to protect against cold injury and ischemic damage. The intravenous PEG 35 administration by a unique dose protected steatotic rat liver grafts against the deleterious effects of cold storage and the subsequent reperfusion. The rationale for the intravenous administration of PEG 35 was to induce a pharmacological preconditioning effect; its protective mechanisms are related to preservation of mitochondria and the induction of protective cell signaling pathways (eNOS, Akt, and AMPK).

An additional model of liver preconditioning by pharmacological induction of adenosine A2a receptor (A2aR) was reported by E. Alchera et al. The analysis of the molecular changes induced by A2aR stimulation revealed multiple mechanisms of liver cell protection that can be both immediate (early preconditioning) and delayed (late preconditioning). Note that the A2aR activation also protects against lipotoxicity by preventing lipoapoptosis in primary rat hepatocytes.

Among the hepatic mechanisms involved in the development of I/R, mitochondrial dysfunction represents a crucial cellular event contributing to I/R injury. As reported by K. Go et al., the elimination of abnormal and dysfunction mitochondria by mitophagy appears to increase both the quality of the mitochondria and cell survival. Although the mechanisms underlying the onset and propagation of mitophagy remain elusive, this event represents an early adaptive response to facilitate cell survival during I/R.

Articles published in this issue not only help to identify new pharmacological approaches for improving liver function during ischemia/reperfusion damage but also stimulate the identification of molecular mediators of hepatic injury.

*Mariapia Vairetti* *Hartmut Jaeschke* *Diethard Monbaliu* *Jae-Sung Kim* *Andrea Ferrigno*

